# Post-graduate CME

**DOI:** 10.4103/0019-5545.42403

**Published:** 2008

**Authors:** Chittaranjan Andrade

**Affiliations:** Department of Psychopharmacology, National Institute of Mental Health and Neurosciences, Bangalore - 560 029, India

## CME QUESTIONS

(A) Weight gain is a vexing problem with many psychotropic drugs, especially atypical antipsychotics such as clozapine and olanzapine. With this background, mark *True* or *False* against each of the following statements:

1. Reboxetine attenuates weight gain in patients who begin treatment with olanzapine.
2. Metformin prevents weight gain in patients who begin treatment with olanzapine.
3. Metformin promotes weight loss in patients who gain weight with olanzapine.
4. Rimonabant promotes weight loss in patients who gain weight with olanzapine.

(B) There are several reasons why psychiatrists often see diabetic patients: diabetes is associated with an increased risk of depression; psychiatric disorders may be associated with hypercortisolemia and other neurohormonal changes that can worsen diabetes; and many medications used in psychiatry may precipitate or worsen diabetes.

The glycated hemoglobin level is a useful measure of blood sugar control during the past 1-3 months; normal levels lie in the 4-6% range. Intensive diabetic control seeks to keep the glycated hemoglobin level within the normal range or, at any rate, below 6.5%. With this background, mark *True* or *False* against each of the following statements:

1. Intensive diabetic control is associated with a lower risk of ischemic cardiovascular or cerebrovascular events.
2. Intensive diabetic control is associated with a lower risk of mortality.
3. Intensive diabetic control is associated with a lower risk of nephropathy.
4. Intensive diabetic control is associated with a higher risk of hypoglycemic events.

(C) In 2005, the Food and Drug Administration (FDA) in the United States issued an alert that, among elderly patients with dementia, the treatment of behavioral disorders with atypical antipsychotic drugs is associated with a higher mortality rate. With this background, mark *True* or *False* against each of the following statements:

1. Recent epidemiological studies suggest that the FDA alert was unjustified.
2. Atypical antipsychotic drugs increase the mortality risk in elderly subjects with dementia.
3. Elderly subjects with dementia who require antipsychotic drugs have a form of illness that is associated with a worse prognosis, including a higher mortality risk.
4. Relative to the atypical drugs, typical antipsychotics carries a lower mortality risk in elderly subjects with dementia.

## CME ANSWERS

### (A) Weight gain with olanzapine

Answers: 1. True; 2. False; 3. True; 4. False.

## DISCUSSION

At least two studies[1,2] have shown that reboxetine (4 mg/day), but not fluoxetine[3] attenuates (but does not prevent) weight gain in schizophrenic patients who begin treatment with olanzapine. In other words, patients prescribed olanzapine do gain weight despite reboxetine, but not as much weight had they not taken reboxetine.

Sibutramine, orlistat, and rimonabant are approved treatments for promoting weight loss; of these, only sibutramine (10-15 mg/day) has been studied, with positive results, in patients who gained weight with olanzapine.[4] There are no studies examining the use of rimonabant in the context of weight gain in schizophrenia. Other treatments which promote weight loss in those who gain weight with olanzapine include topiramate[5] and amantadine.[6]

Several studies have shown that, in children and adolescents as well as in adults who receive olanzapine or other psychotropic drugs, metformin (750-1500 mg/day) attenuates (but does not prevent) weight gain and promotes weight loss. The drug is effective and well tolerated in studies that extend for up to 16 weeks,[7–12] though, found no benefit with metformin.

Would the discontinuation of the effective medications result in a loss of the accrued benefits? What is the long-term safety and efficacy of drugs prescribed for weight loss in psychiatric patients? Both questions require research. It should be kept in mind that B12 and folate deficiency may develop in chronic users of metformin, and that metformin may also result in fatal lactic acidosis in patients with certain medical disorders.[13]

### (B) Intensive control of blood sugar

Answers: 1. False; 2. False; 3. True; 4. True.

## DISCUSSION

Outcomes with intensive blood sugar control were investigated in the ACCORD (*n* = 10,251) and ADVANCE (*n* = 11,140) randomized controlled trials. These studies found that, in patients with type 2 diabetes mellitus, a glycated hemoglobin target of <6.5% reduced the risk of nephropathy but not the risk of adverse cardiovascular and cerebrovascular events, nor the risk of retinopathy. Alarmingly, intensive blood sugar control increased the risk of hypoglycemic events and, in the ACCORD study, also increased the risk of mortality.

These studies differed much in their methods, as a result of which the interpretation of results is a complex matter. Nonetheless, neither study clearly encourages the use of intensive treatment regimens for diabetic patients. Interested readers are referred to the excellent commentaries on these two trials.[14,15]

### (C) Risks with antipsychotic medications in dementia

Answers: 1. False; 2. True; 3. True; 4. False.

## DISCUSSION

Two large epidemiological studies examined the risk of serious adverse events and death associated with atypical antipsychotic prescriptions in elderly subjects with dementia. In a retrospective study of 10,615 elderly patients with dementia, Kales *et al.*[16] observed that patients started on conventional, atypical, or both categories of antipsychotic medications had a 23-29% one-year mortality rate whereas those started on other psychiatric medications had only a 15% one-year mortality rate. The mortality risk with typical antipsychotics was similar to that with the atypical drugs. Interestingly, the higher mortality rates in patients taking antipsychotic medications appeared to be due to dementia-related causes; this implies that dementia necessitating antipsychotic treatment may be intrinsically associated with a worse prognosis.

In a retrospective study of 41,241 elderly patients with dementia, Rochon *et al.*[17] observed that the 1-month risk of a serious adverse event or death was two to three times higher in patients receiving antipsychotic medication relative to those not prescribed antipsychotic medication. The risk with conventional antipsychotics was slightly higher than that with the atypical drugs.

Studies such as these cannot attribute risk causality to antipsychotic medication. Causality was shown by a meta-analysis of 15 randomized, placebo-controlled trials;[18] that mortality was significantly higher in dementia patients who received antipsychotic medication than in those who received placebo (3.5% *vs*. 2.3%, respectively).
